# Clinical Lecture on Pneumothorax

**Published:** 1896-04-11

**Authors:** S. H. Habershon

**Affiliations:** Assistant Physician and Pathologist to the Brompton Hospital for Consumption and Diseases of the Chest


### Medical Progress and Hospital Clinics.

[*The Editor will be glad to receive offers of co-operation and contributions from members of the profession. All letters should be addressed to* The Editor, at the Office, 428, Strand, London, W.C.]

### CLINICAL LECTURE ON PNEUMOTHORAX.

By S. H. Habershon, M.D., F.R.C.P., Assistant Physician and Pathologist to the Brompton Hospital for Consumption and Diseases of the Chest.

### I.—The Causes of Pneumothorax.

By pneumothorax we mean the presence of air or gas in the pleural cavity. The serous membrane lining the pleural cavity is somewhat tough and elastic, and it is evident that a breach of surface can only take place from the accidental formation of an opening in the visceral or costal pleura, by which the free ingress of the external air is permitted. We know of no disease, except, perhaps, a suppurating hydatid, in which fluid enclosed in an airtight cavity has the power of generating gas, and such a condition may be, therefore, left out of account.

In defining the causes that tend to produce a rupture or perforation of the pleural membrane, we may first take the external layer, including not only the costal portions of the pleura but also the diaphragmatic layer and the pleura reflected over the œsophagus, or contiguous to it, and next rupture cf the visceral layer.

1. **Rupture of the External Pleural Layers.**—The rupture may be (*a*) traumatic—from a wound of the chest wall. In rare cases Fagge states that as a result of tracheotomy interstitial emphysema may spread from the situation'of the wound, and, extending along the root of the lung, rupture into both pleural cavities, producing a double pneumothorax. I need scarcely say that this must necessarily be exceedingly rare. (*b*) From perforation of the diaphragm by a subdiaphragmatic abscess; this has been known to occur as a result of perforation of the stomach or duodenum, and from a suppurating hydatid cyst of the liver; or (*c*) from perforation of the œsophagus from the ulceration of a malignant growth. All these cases have been reported in the various medical journals and papers.

2. **Perforation of the Visceral or Pulmonary Layer of the Pleura from within.**—Inflammations of the pleura, as a rule, tend to cause thickening and adhesion, but in some cases suppurative inflammation may lead to sloughing of the visceral pleura. Perforation may be caused by—(*a*) The bursting of an empyema through the pleura into a bronchus (this is by no means an uncommon accident); or (*b*) may be traumatic, *e.g.*, a wound of the lung from a fractured rib.

3. **Rupture or Perforation of the Visceral Layer of Pleura from Disease of the Lungs.**—This cause includes by far the larger number of cases. In a few instances the healthy pleura has been said to be ruptured by violent strain or muscular exertion, as mentioned by Fagge in a case recorded in Ziemssen's "Encyclopædia," but this must be exceedingly rare in the absence of some disease weakening the pleura. This class includes—(*a*) Abscess, necrosis, or gangrene of the superficial portion of the lung. Most of these cases are due to tubercular disease, and we shall, therefore, deal fully with the pathology of this affection. Gangrene or abscess of the lung may result as a sequela of pneumonia. Septic abscesses of the lung may cause pneumothorax, as in one of two cases recently reported (on February 14th) at the Clinical Society during the course of typhoid fever. Sloughing infarcts or embolic abscesses have also been said to perforate the pleura. (*b*) Rupture of an emphysematous bleb or of the saccules of a dilated bronchial tube. This occurs in rare instances during the course of phthisis, and I am able to show you two specimens. One of my cases—a very remarkable one—is a case of primary hydropneumothorax. (*c*) Hydatid disease of the lung. This has several times occurred, and is the cause of a primary hydropneumothorax, the appearance of serous fluid coinciding with the occurrence of a pneumothorax. In one of the specimens I have to show this combination occurred, but the disease was due to a localised tubercular focus.

Of all these varied causes the most frequent is that of tubercular disease of the lung; and I shall now proceed to discuss the conditions under which it is liable to occur. It is said that tubercular disease is responsible for no less than 90 per cent. of the cases of pneumothorax, and though some of the other causes have occurred in this hospital—mainly through the bursting of an empyema, the rupture of an emphysematous bleb, or the formation of a perforating hydatid abscess—still in a consumption hospital I shall be pardoned for laying the greatest stress upon this class of cases that come so frequently under our notice. Between the years 1885 and 1896 (the present date) about 1,375 post-mortems have been performed on cases of phthisis alone (about 400 of which are my own). During this period 114 cases of pneumothorax have occurred amounting to a trifle over 8 per cent. of all the cases.

Post-mortems on Tubercular Disease of the Lungs in the Hospital for Consumption, Brompton.

| Year. | Cases of Phthisis. | Pneumothorax. | Year. | Cases of Phthisis. | Pneumothorax. |
|---|---|---|---|---|---|
| 1885 | 88 | 5 | Brought forward | 717 | 52 |
| 1886 | 91 | 12 | 1892 | 208 | 16 |
| 1887 | 72 | 9 | 1893 | 174 | 14 |
| 1888 | 77 | 7 | 1894 | 146 | 16 |
| 1889 | 114 | 5 | 1895 | 115 | 13 |
| 1890 | 114 | 7 | 1896 (to Feb. 18) | 15 | 3 |
| 1891 | 161 | 7 | | | |
| Carried forward | 717 | 52 | Total | 1,375 | 114 |

It is the rule in phthisis that the tubercular affection of lung is accompanied by pleurisy, which causes the overlying visceral pleura to become adherent to the costal surface. In chronic cases this adhesion is extremely firm and associated with increase in thickness and density, so that the two layers ultimately become inseparable. If it were not for this fact pneumothorax would be an extremely common accident. The process of ulceration of the lung frequently lays bare the surface of the pleura, hence the adhesion becomes a great protection. When the whole lung is affected with chronic disease a pneumothorax is extremely rare. What does happen, and that not infrequently, is that a sinus is formed, just as in the case of any other abscess cavity, which may even perforate the adherent layers of pleura and ulcerate the rib, or eventually find its way to the skin or even through the skin. Two cases I shall show. In one a perforation of both layers of pleura has taken place with ulceration of the rib; in the other, two of the ribs are extremely necrosed, and nothing but skin overlies the cavity. The pleural layers have, however, been partly separated, and in consequence a small local pneumothorax has resulted.

Pneumothorax occurs in acute cases, or even in a chronic case, from the acute softening or ulceration of lung in a portion of one of the lobes situated below the part affected with the chronic disease. In the first class of cases it is usually in the broncho-pneumonic variety which proceeds rapidly to caseation and softening, often too rapidly for intimate and firm adhesion of the pleura to take place. In a few cases there may be a very small focus of disease in the lung (and this totally unsuspected before the occurrence of pneumothorax), but, unfortunately for the patient, this limited patch of tubercular caseation and necrosis has been situated immediately on the surface of the lung, frequently in the lower part of the upper lobe or on the apex of the lower lobe. Two cases of this condition are shown. The commoner class is the second, in which the apex of the lung has been the site of subacute or chronic disease, and a more rapid or acute spread has taken place in the lower part of the upper lobe or in the lower lobe. This is the reason why the aperture of a pneumothorax is so commonly found in the middle third of the lung, often in the lateral portion situated about the third or fourth intercostal spaces, and almost invariably immediately below the line of strongly adherent layers of pleura. The opening itself may be single, small, pinpoint, valvular, or a narrow slit. On the other hand, it is common to find several circular or irregular apertures, and in some cases an extensive area of visceral pleura has sloughed away. In many cases other thinned spots of pleura almost on the point of rupturing, and overlying necrotic areas of lung, are found. The edges of the opening are frequently covered with plastic lymph, and the rest of the pleura is usually the seat of an acute pleurisy or covered with a buttery layer of lymph. In chronic cases the pleura may become extremely thickened from the deposit and organisation of lymph on its inner and costal surfaces. The acute pleurisy resulting from pneumothorax is generally, but not always, associated with an effusion of fluid. If the purulent contents of a vomica are discharged into the pleura this effusion is certain to produce a septic condition of the pleural cavity or an acute tubercular outbreak, and a pyopneumothorax results. In other cases where there is reason to believe that the cavity has been small, the effusion may be serous; or, as is frequently proved by the post-mortem records, there is none at all. A serous effusion which results from a small tubercular lesion will, however, become purulent in many cases. It is an extremely rare occurrence to find on a post-mortem table a double pneumothorax. Such a case, however, occurred some days ago, and I am able to show the specimen. Needless to say the result was very speedily fatal, but the accident occurred a few hours before death.

